# Supplementary material for: Predicting in-hospital mortality in ICU patients with lymphoma using machine learning models
Source: PLoS One. 2025 Aug 20;20(8):e0330197. doi: 10.1371/journal.pone.0330197 (PMC12367167; doi:10.1371/journal.pone.0330197)
Supplement: S1 Table — (DOCX) [file pone.0330197.s002.docx]

Table S1. Extent of missing data before imputation (n = 1 591)

| Variable | Miss.freq | Miss.percentage% |
| --- | --- | --- |
| gender | 0 | 0 |
| age | 0 | 0 |
| heart_rate | 5 | 0.3143 |
| sbp | 11 | 0.6914 |
| dbp | 11 | 0.6914 |
| temperature | 35 | 2.1999 |
| spo2 | 6 | 0.3771 |
| hematocrit | 15 | 0.9428 |
| hemoglobin | 15 | 0.9428 |
| platelets | 17 | 1.0685 |
| wbc | 19 | 1.1942 |
| aniongap | 17 | 1.0685 |
| bicarbonate | 17 | 1.0685 |
| bun | 17 | 1.0685 |
| calcium | 68 | 4.274 |
| chloride | 17 | 1.0685 |
| creatinine | 16 | 1.0057 |
| glucose | 20 | 1.2571 |
| sodium | 18 | 1.1314 |
| potassium | 17 | 1.0685 |
| inr | 158 | 9.9309 |
| pt | 158 | 9.9309 |
| aptt | 176 | 11.0622 |
| myocardial_infarct | 0 | 0 |
| heart_failure | 0 | 0 |
| peripheral_vascular | 0 | 0 |
| dementia | 0 | 0 |
| cerebrovascular | 0 | 0 |
| chronic_pulmonary_disease | 0 | 0 |
| rheumatic_disease | 0 | 0 |
| peptic_ulcer_disease | 0 | 0 |
| mild_liver_disease | 0 | 0 |
| diabetes | 0 | 0 |
| paraplegia | 0 | 0 |
| renal_disease | 0 | 0 |
| malignant_cancer | 0 | 0 |
| severe_liver_disease | 0 | 0 |
| metastatic_solid_tumor | 0 | 0 |
| aids | 0 | 0 |
| gender | 0 | 0 |
| mor_hospital | 0 | 0 |
