# Supplementary figures and images for: Predicting in-hospital mortality in ICU patients with lymphoma using machine learning models
Source: PLoS One. 2025 Aug 20;20(8):e0330197. doi: 10.1371/journal.pone.0330197 (PMC12367167; doi:10.1371/journal.pone.0330197)

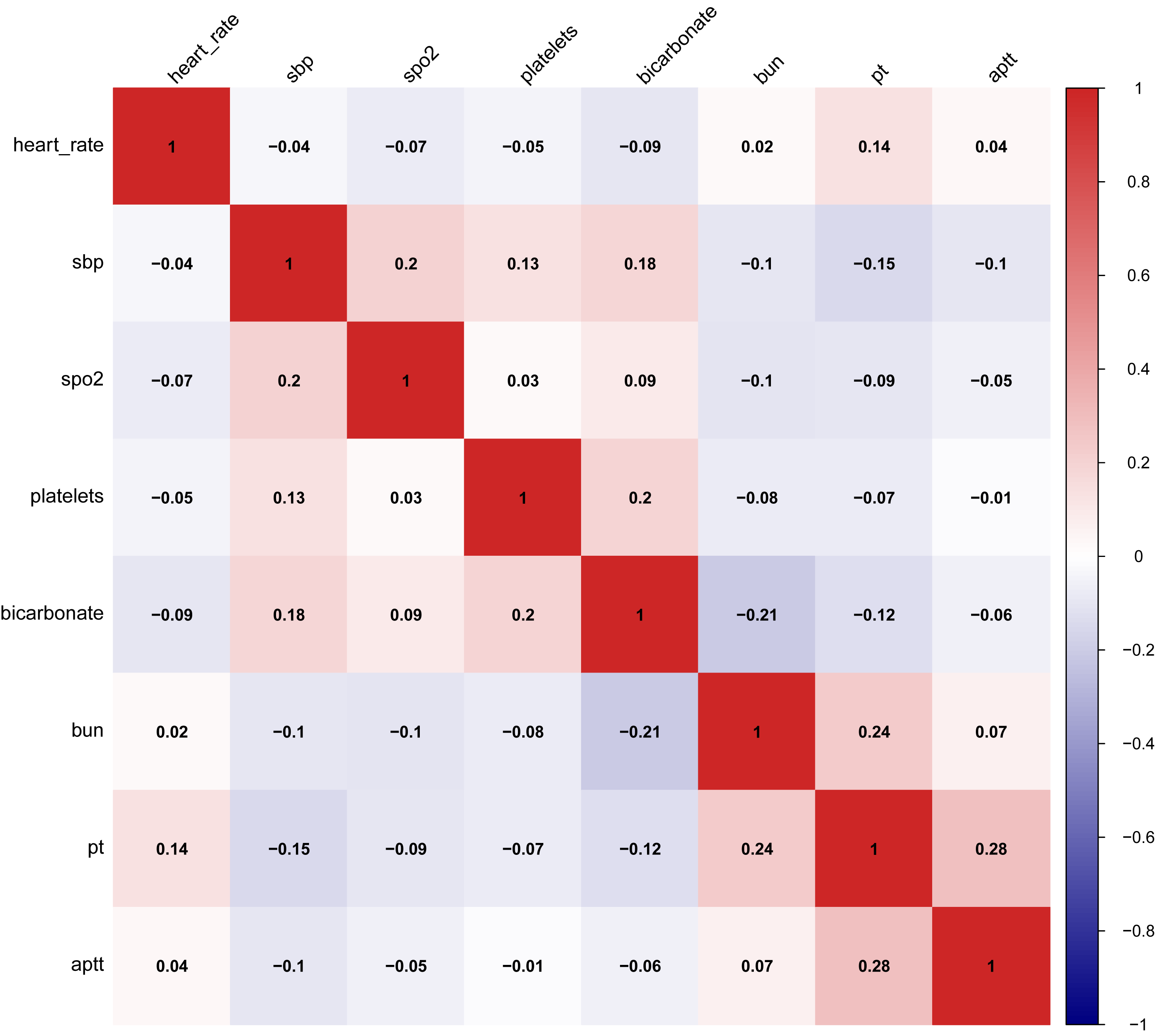

Supplement: S1 Fig — (TIF) [file pone.0330197.s003.tif]
