# Supplementary material for: Predicting in-hospital mortality in ICU patients with lymphoma using machine learning models
Source: PLoS One. 2025 Aug 20;20(8):e0330197. doi: 10.1371/journal.pone.0330197 (PMC12367167; doi:10.1371/journal.pone.0330197)
Supplement: Attachment — Submitted filename: Response to Reviewers.docx [file pone.0330197.s005.docx]

Journal Requirements:

When submitting your revision, we need you to address these additional requirements.

**1. Please ensure that your manuscript meets PLOS ONE's style requirements, including those for file naming. The PLOS ONE style templates can be found at**

**https://journals.plos.org/plosone/s/file?id=wjVg/PLOSOne_formatting_sample_main_body.pdf and https://journals.plos.org/plosone/s/file?id=ba62/PLOSOne_formatting_sample_title_authors_affiliations.pdf**

**Response:**

Thank you for reminding us of PLOS ONE’s formatting and file-naming standards.

We have revised the manuscript and supplementary files to fully comply with PLOS ONE’s style requirements, including file naming, formatting, figure preparation, and ethical/data-availability statements.

**2. Please note that PLOS ONE has specific guidelines on code sharing for submissions in which author-generated code underpins the findings in the manuscript. In these cases, all author-generated code must be made available without restrictions upon publication of the work. Please review our guidelines at https://journals.plos.org/plosone/s/materials-and-software-sharing#loc-sharing-code and ensure that your code is shared in a way that follows best practice and facilitates reproducibility and reuse.**

**Response:**

Thank you very much for your guidance regarding PLOS ONE’s code-sharing requirements.

We have carefully reviewed PLOS ONE’s code-sharing policy and confirm that all author-generated code that underpins the findings of this study is publicly available without restrictions.

**3. When completing the data availability statement of the submission form, you indicated that you will make your data available on acceptance. We strongly recommend all authors decide on a data sharing plan before acceptance, as the process can be lengthy and hold up publication timelines. Please note that, though access restrictions are acceptable now, your entire data will need to be made freely accessible if your manuscript is accepted for publication. This policy applies to all data except where public deposition would breach compliance with the protocol approved by your research ethics board. If you are unable to adhere to our open data policy, please kindly revise your statement to explain your reasoning and we will seek the editor's input on an exemption. Please be assured that, once you have provided your new statement, the assessment of your exemption will not hold up the peer review process.**

**Response:**

Thank you for the editorial reminder. We have now finalized our data-sharing plan and confirm that all datasets analysed in the study will be made freely accessible upon acceptance. Specifically:

• The fully de-identified MIMIC-IV v3.1 database can be obtained by any credentialed researcher who completes the CITI “Data or Specimens Only Research” course and signs the PhysioNet Data Use Agreement (https://physionet.org/content/mimiciv/3.1/).

• No identifiable patient information is included; therefore, public release complies with the protocol approved by our institutional review boards and does not breach any ethics requirements.

We have updated the “Data Availability” statement in the revised manuscript accordingly and confirm that no exemption from open-data sharing is requested.

**4. Your ethics statement should only appear in the Methods section of your manuscript. If your ethics statement is written in any section besides the Methods, please move it to the Methods section and delete it from any other section. Please ensure that your ethics statement is included in your manuscript, as the ethics statement entered into the online submission form will not be published alongside your manuscript.**

**Response:**

We thank the editors for pointing this out. In the revised manuscript we have moved the complete ethics statement into the Methods section under the subheading “Data Source and Study Design,” and we have removed any duplicate or redundant ethics language from all other sections (including the Acknowledgements). The manuscript now contains a single, concise ethics statement that confirms the use of de-identified public data (MIMIC-IV), exemption from IRB review, and compliance with the 1964 Helsinki Declaration and its later amendments.

**Reviewer #1:**

**1. Regarding the outcome measure, while the study designates "in-hospital mortality" as the primary endpoint, further clarification on the temporal boundary of this definition would enhance the clinical interpretability of results. Given that mortality at different time points in ICU patients carries distinct clinical implications (e.g., 7-day mortality reflecting acute intervention efficacy, 28-day mortality indicating comprehensive treatment outcomes, and long-term mortality associated with underlying disease progression), it is recommended to reference the standard definitions in comparable studies and supplement the methodological rationale for time frame delineation.**

**Response:**

We thank the reviewer for this valuable comment. In the revised Methods section, under “Outcome Definition,” we have now added explicit temporal boundaries:

In-hospital mortality is defined as death occurring at any time between ICU admission and hospital discharge (index hospitalisation), consistent with the standard definition adopted by the MIMIC-IV database and prior large-scale ICU outcome studies (Noritomi et al., 2021; Rajkomar et al., 2018). This endpoint was chosen because (1) it captures the full spectrum of acute and subacute mortality risk directly pertinent to ICU prognostication, and (2) it aligns with clinical decision-making contexts where treatment intensity and goals-of-care discussions are re-evaluated throughout the entire hospital stay. Although 7-day or 28-day mortality are alternative metrics, they were not selected because ICU length of stay in lymphoma patients is often prolonged by chemotherapy schedules and infectious complications, making in-hospital mortality the most clinically relevant and patient-centred outcome for this cohort.

**2. Potential collinearity exists among variables selected by Lasso regression (e.g., BUN and Cr as renal function markers, platelet count and PT in coagulation function). Although Lasso regularization mitigates this issue, adding a correlation matrix would strengthen the argument for model robustness. Additionally, incorporating SHAP dependence plots or interaction value analyses to explore nonlinear interactions between key variables (e.g., BUN and platelet count) could provide richer evidence for clinical interpretation of model prediction mechanisms.**

**Response:**

We thank the reviewer for highlighting the importance of demonstrating the absence of harmful collinearity. In the revised manuscript we have added a full Pearson correlation matrix for the eight LASSO-selected variables (Supplementary Figure S1). The highest absolute correlation observed is 0.60 (BUN vs. creatinine), followed by 0.40 (platelets vs. PT); all remaining pairwise correlations are < 0.30. These low-to-moderate values indicate that multicollinearity is not a concern after LASSO regularisation, and the model’s coefficients remain stable and interpretable.

Given the limited magnitude of these correlations and the fact that the LASSO penalty already shrinks highly collinear predictors, we did not deem SHAP interaction analyses essential. Instead, the correlation matrix and the robust performance metrics (AUC = 0.7766, stable across cross-validation folds) jointly support the model’s reliability.

**3. While baseline characteristics acknowledge the impact of multiple comorbidities, integrating a standardized comorbidity index (e.g., Charlson Index) to quantify comorbidity burden would systemize the risk factor analysis. As different comorbidities contribute differently to mortality, supplementing correlation analyses between comorbidity indices and death outcomes would enhance the clinical relevance of findings.**

**Response:**

We appreciate the reviewer’s suggestion. After LASSO-based feature selection, none of the individual comorbidity indicators were retained in the final eight-variable model. Because the Charlson Comorbidity Index (CCI) itself is a composite of those same comorbidities, including it would introduce redundancy and violate the parsimony principle already enforced by LASSO. Furthermore, when we compared the predictive performance of (a) the LASSO-selected model versus (b) the same model augmented with the CCI, the AUC remained essentially unchanged (ΔAUC = +0.003). Therefore, we elected to keep the original, more parsimonious set of variables and did not integrate the Charlson Index.

**Reviewer #2:** Its my pleasure to review the manuscript titled "Predicting In-Hospital Mortality in ICU Patients with lymphoma Mellitus Using Machine Learning Models". The study develops and validates ML models for predicting in-hospital mortality in ICU patients with presumed lymphoma. While the methodology is generally sound and addresses a clinically relevant problem, critical terminology errors undermine the foundation of the work. Significant revisions are required before consideration for PLOS ONE. The AUC of 0.7766 is modest, and clinical applicability needs stronger justification.

________________________________________

**The major Issues of the research included:**

**1. "Lymphoma Mellitus" is incorrect and non-existent. "Mellitus" specifically refers to diabetes (e.g., Diabetes Mellitus). The correct term is simply "Lymphoma".**

**Response:**

We sincerely thank the reviewer for pointing out the terminology error. We fully acknowledge that “Lymphoma Mellitus” is an incorrect and non-existent term; “mellitus” is reserved for diabetes mellitus. In the revised manuscript we have carefully replaced every instance of “Lymphoma Mellitus” with “Lymphoma” throughout the title, abstract, main text, figures, tables, and supplementary materials to ensure accuracy and clarity.

**2. The introduction could better emphasize the specific challenges of predicting mortality in lymphoma patients compared to the general ICU population (e.g., unique complications like tumor lysis syndrome, immunosuppression-related infections, specific organ involvement). The gap regarding ML for this specific subgroup is adequately stated.**

**Response:**

We appreciate the reviewer’s helpful suggestion. In the revised Introduction (paragraph 2, lines 45–58) we have added a concise paragraph that explicitly contrasts lymphoma ICU patients with the general ICU population. We now highlight lymphoma-specific mortality drivers such as acute tumor lysis syndrome, profound immunosuppression-related opportunistic infections (e.g., Pneumocystis jirovecii, invasive moulds), chemotherapy-induced cardiotoxicity, and direct renal or hepatic infiltration. These unique pathophysiological features render traditional scores (SOFA, APACHE) less reliable for this subgroup, underscoring the need for tailored machine-learning models.

**3. The exclusion of 10,054 patients due to "missing values for all variables" (Fig 1) is unusual and concerning. It suggests potential selection bias. Were these patients truly missing every single variable collected? Clarification or rephrasing is needed (e.g., "missing key predictor or outcome variables").**

**Response:**

We thank the reviewer for raising this important point. The wording in the original flow-chart was indeed imprecise. The 10 054 patients were not missing every variable; rather, they were excluded because they lacked one or more of the key predictor or outcome variables required for modelling (e.g., vital signs at ICU admission, essential laboratory values such as BUN or platelets, or the in-hospital mortality flag). These missing data rendered them non-evaluable by our modelling pipeline. We have updated the legend of Figure 1 and the Methods section (Data Collection and Processing) to clarify this exclusion criterion as “missing key predictor or outcome variables” instead of the overly broad phrase “missing values for all variables.” This change more accurately reflects the selection process and mitigates concern for undue selection bias.

**4. The long timeframe (2008-2019) introduces potential confounding from evolving ICU practices and lymphoma treatments over 11 years. This isn't addressed.**

**Response:**

We appreciate the reviewer’s concern regarding the 2008–2019 time span and its potential for confounding by evolving ICU practices and lymphoma treatments. Because the MIMIC-IV database does not contain detailed, time-stamped information about chemotherapy regimens, immunotherapy protocols, or changes in critical-care bundles over the years, we are unable to adjust for these factors in the current analysis. Consequently, we have added the following sentence to the Limitations section of the revised manuscript:

“Given the absence of granular treatment data (e.g., specific chemotherapy agents, immunotherapies, or evolving ICU bundles) in MIMIC-IV, we could not control for temporal changes in lymphoma management or critical-care practices. Future multicenter studies that incorporate longitudinal drug and intervention data are warranted to address this limitation.”

**5. MIMIC-IV, while large, is single-center data (Beth Israel Deaconess MC). Generalizability to other settings may be limited, appropriately noted as a limitation.**

**Response:**

We thank the reviewer for highlighting the issue of generalizability related to the single-center nature of MIMIC-IV. We fully agree that the database is derived solely from Beth Israel Deaconess Medical Center (Boston, USA) and may not reflect patient demographics, ICU practices, or lymphoma treatment patterns in other settings. In the revised manuscript we have explicitly stated:

“MIMIC-IV is a single-center database derived from Beth Israel Deaconess Medical Center (Boston, USA), which may introduce selection bias and limit generalizability to other ICUs with different patient demographics, clinical practices, or resource availability. Future research should conduct external validation on multiple independent datasets to confirm the generalizability of the models.”

**6. While LASSO identified predictors, the justification for the initial set of variables extracted from MIMIC-IV is somewhat brief. Were lymphoma-specific variables (e.g., disease stage, type [Hodgkin/Non-Hodgkin], recent chemotherapy, presence of tumor lysis syndrome, neutropenia) considered or available? These are highly relevant to mortality risk in lymphoma patients.**

**Response:**

We thank the reviewer for emphasizing the clinical importance of lymphoma-specific covariates. Unfortunately, MIMIC-IV does not contain ICD-O histology codes, Ann Arbor stage, Hodgkin vs. non-Hodgkin sub-typing, date-stamped chemotherapy records, tumour-lysis-syndrome flags, or serial neutrophil counts that would allow reliable identification of neutropenia. Consequently, disease stage, lymphoma subtype, recent cytotoxic therapy, TLS, and neutropenia could not be incorporated into the initial variable set. We have now added the following sentence to the Data Collection subsection (Methods) and to the Limitations section:

“Because MIMIC-IV lacks lymphoma-specific variables such as histological subtype, Ann Arbor stage, recent chemotherapy exposure, tumour-lysis syndrome, or neutrophil nadir, these high-risk features could not be included in the analysis; future multicenter studies with dedicated oncology-ICU databases are needed to capture these predictors.”

**7. The models only use admission/early ICU data. Interventions during the ICU stay (e.g., mechanical ventilation, vasopressors, dialysis, specific lymphoma treatments) are not included as potential predictors or confounders, significantly limiting the model's potential clinical applicability for dynamic risk assessment. This is a major omission.**

**Response:**

We thank the reviewer for pointing out this critical limitation. MIMIC-IV does not contain granular, time-stamped logs of mechanical-ventilator settings, vasopressor doses, renal-replacement-therapy sessions, or lymphoma-specific therapies (e.g., rasburicase, dose-adjusted chemotherapy) administered after ICU admission. Consequently, these dynamic interventions could neither be included as predictors nor adjusted for as potential time-varying confounders, which indeed curtails the model’s utility for real-time risk updating. We have amended the Limitations section to read:

“Because MIMIC-IV lacks detailed, time-stamped data on ICU interventions (mechanical ventilation, vasopressors, dialysis, or lymphoma-specific treatments administered after admission), our models are restricted to admission/early-ICU variables and cannot perform dynamic risk reassessment. Future linkage with high-resolution treatment logs is required to develop longitudinal prediction frameworks.”

**8. The P-values in Table 1 require context. With 1591 patients, very small differences can become statistically significant. Emphasis should be on clinically meaningful differences. Reporting effect sizes (e.g., mean difference, Cohen's d for continuous; odds ratio for categorical) alongside P-values would be beneficial. Some variables (e.g., platelets, BUN) show large and clinically meaningful differences.**

**Response:**

We appreciate the reviewer’s concern that, with 1 591 patients, even trivial absolute differences may reach statistical significance.

1. Table 1 is intended solely as a descriptive summary of baseline characteristics to demonstrate face validity of the two groups; it is not designed to make causal inferences. Therefore, we have not calculated formal effect-size metrics such as Cohen’s d or odds ratios.

2. To help readers distinguish statistical from clinical importance, we have now added a cautionary sentence to the footnote of Table 1:

“Because this table is descriptive, effect sizes and odds ratios were not computed. Readers should weigh the absolute differences in the context of clinical relevance rather than relying on P-values alone.”

3. Any clinically relevant imbalances identified in Table 1 will be appropriately adjusted for in the multivariable analyses presented later in the manuscript, thereby limiting the risk of over-interpretation of these baseline comparisons.

**9. Details on hyperparameter tuning for the ML models (especially complex ones like CatBoost, NN) are lacking. Was tuning performed? How? This impacts performance and reproducibility.**

**Response:**

We thank the reviewer for this insightful comment. In the revised manuscript we now provide a detailed description of the hyper-parameter tuning procedure for every model, especially for the more complex ones.

All 15 algorithms were tuned exclusively within the 70 % training set using a 5-fold stratified nested cross-validation scheme driven by Bayesian optimisation (Optuna, 100 trials, AUC as the objective). The test set remained untouched throughout the search to avoid any information leakage.

•CatBoost: learning_rate (0.01–0.3), depth (4–10), l2_leaf_reg (1–9), bagging_temperature (0–1), and class-weight strategy.

• Neural network (MLP): hidden-layer sizes (64–512 neurons, 1–3 layers), dropout (0–0.5), batch size (32–256), learning-rate schedules (Adam, RMSprop), and L2-regularisation (1e-5–1e-2).

• XGBoost, LightGBM, Random Forest, and Extra Trees received comparable ranges for depth, learning_rate/eta, min_child_samples, subsample, colsample_bytree, and class weights.

The best hyper-parameters were locked, the final model was re-fitted on the full 70 % training set, and evaluated once on the untouched 30 % test set.

We sincerely appreciate the reviewer’s careful review and constructive feedback.

**10. The best model's AUC (0.7766) is modest for a clinical prediction model. While potentially better than traditional scores (though direct comparison isn't rigorously made), an AUC <0.8 often indicates limited clinical utility for individual prediction. This needs careful interpretation and tempering of claims about "high predictive performance" or "significant outperforming".**

**Response:**

We thank the reviewer for highlighting the need for cautious interpretation of our AUC. In the revised manuscript we have:

1. Replaced the phrase “high predictive performance” with the more accurate “moderate discriminative ability.”

2. Explicitly stated that an AUC of 0.776 (95 % CI 0.741–0.811) is below the commonly cited 0.80 threshold for high clinical utility at the individual-patient level.

3. Clarified that the model’s value lies mainly in (a) outperforming the generic SOFA/APACHE scores in a head-to-head comparison on the same cohort (ΔAUC ≈ +0.05–0.07, DeLong p < 0.01), and (b) providing transparent, SHAP-based risk explanations that may aid—not replace—clinical judgment.

4. Added a limitation paragraph noting that the modest AUC underscores the need for external validation and integration of lymphoma-specific predictors (e.g., tumour lysis markers, recent chemotherapy) before any bedside deployment.

We have tempered all claims accordingly and thank the reviewer again for this constructive feedback.

**11. The description of data splitting for training/validation is unclear. Was a strict hold-out test set used after feature selection (LASSO) and hyperparameter tuning? Or was everything done within cross-validation folds? Preventing data leakage is crucial; the methodology section needs clarification.**

**Response:**

We appreciate the reviewer’s concern about potential data leakage. In the revised Methods section we now provide a step-by-step, unambiguous description of the data-splitting procedure:

1. Single, stratified split before any model development

• The entire cohort (n = 1 591) was divided once—stratified by the in-hospital mortality label—into a 70 % training set (n = 1 114) and a strictly untouched 30 % hold-out test set (n = 477).

• No observation from the test set was ever used for feature selection, hyper-parameter tuning, or model training.

2. Nested procedure within the training set

• Feature selection (LASSO) and Bayesian hyper-parameter optimisation (100 trials) were performed exclusively inside the 70 % training data, using an inner 5-fold cross-validation loop.

• After the optimal LASSO feature subset and hyper-parameters were fixed, the CatBoost classifier was retrained on the full 70 % training set and evaluated exactly once on the 30 % hold-out set to obtain the reported metrics.

3. Prevention of leakage for imputation and class imbalance

• Multiple imputation by chained equations (MICE) was fitted on the training set; the imputation models were then applied to the test set without refitting.

• Handling of class imbalance (class weights or RandomOverSampler) was likewise learned only on the training folds and applied to the untouched test set.

These details have been added verbatim to the revised Methods, ensuring complete transparency and adherence to best-practice leakage-prevention standards.

**12. The class imbalance (21.5% mortality) is acknowledged but the specific techniques used to handle it during model training (e.g., class weighting, sampling methods) are not described. This can significantly impact model performance, especially for metrics like F1-score.**

**Response:**

We thank the reviewer for pointing out the omission. In the revised Methods section we have added a dedicated paragraph that clearly describes how the positive-class imbalance (21.5 % mortality) was addressed during model training and evaluation:

1. Treatment strategy for each algorithm

• Models that natively support class weights (CatBoost, Logistic, Ridge, SVM, LightGBM, XGBoost, Gradient Boosting) were trained with an inverse-frequency weight w = Nmajority / Nminority ≈ 3.65 incorporated into their loss functions.

• Models that do not expose class-weight parameters (Random Forest, Extra Trees, AdaBoost, MLP, Naïve Bayes, KNN, Decision Tree) were trained after applying RandomOverSampler within each cross-validation fold to up-sample the minority class to parity; this resampling was performed exclusively on the training folds and never touched the hold-out test set.

2. Ensuring unbiased evaluation

• All reported metrics (AUC, accuracy, F1-score) were computed on the untouched, imbalanced 30 % test set, thereby reflecting real-world class distribution and avoiding optimistic inflation of the F1-score.

These details have been inserted verbatim into the revised manuscript to ensure full transparency and reproducibility.

**13. Table 1 Presentation: Mixing "mean (SD)" and "Median (IQR)" formats for continuous variables without a clear rationale based on distribution (e.g., normality) is inconsistent. Variables like platelets and BUN are correctly presented as median (IQR) as they are skewed, but others (e.g., heart rate) presented as mean (SD) should be checked for normality or also presented as median (IQR) for consistency. Statistical tests (presumably t-tests/Wilcoxon, Chi-square/Fisher) used for Table 1 are not explicitly named.**

**Response:**

Thank you for drawing attention to the presentation of continuous variables in Table 1. 1. Consistency of format (mean ± SD vs. median [IQR])

Before assembling Table 1 every continuous variable was formally tested for normality (Shapiro–Wilk test). Variables that departed significantly from a normal distribution (p < 0.05) are reported as median [IQR]; those that did not are reported as mean ± SD. Platelets and BUN were indeed skewed and therefore appear as median [IQR], whereas heart rate (as well as other mean ± SD variables) satisfied the normality assumption and are presented as mean ± SD. This approach, based on the result of the normality test rather than on convention alone, is internally consistent and is now explicitly stated in the footnote to Table 1.

2. Explicit statement of statistical tests

• A footnote has been added to Table 1:

“Continuous variables are expressed as mean (SD) if Shapiro-Wilk p ≥ 0.05 and as median (IQR) otherwise. Between-group comparisons used the independent-samples t-test for normally distributed variables, the Mann–Whitney U test for non-normally distributed variables, and the χ² or Fisher exact test for categorical variables.”

These changes ensure consistency in presentation and full transparency of the statistical methods used.

**14. P-value Interpretation: Reliance on P-values <0.05 in Table 1 without adjustment for multiple comparisons (e.g., Bonferroni, FDR) risks false positives. Given the large number of comparisons, discussing clinically significant differences is more important than purely statistical significance.**

**Response:**

We agree that, in isolation, a P-value <0.05 can inflate type-I error when many baseline variables are compared. Table 1 was constructed primarily to display the clinical characteristics of the cohorts and to allow readers to judge face validity; it was not intended to generate etiological conclusions. No adjustment (e.g., Bonferroni or FDR) was applied because:

1. Table 1 is descriptive. The comparisons are hypothesis-generating rather than confirmatory, and the main study question is addressed in the subsequent multivariable model.

2. Adjusting for multiplicity in a descriptive table can obscure clinically meaningful imbalances that still need to be considered when interpreting later analyses.

3. We now explicitly caution readers that statistical significance in Table 1 should not be over-interpreted. A new sentence has been added to the footnote:

“P-values are unadjusted for multiple comparisons; they are provided only to highlight potentially relevant imbalances, not to infer causal differences. Clinical importance should be weighed alongside statistical significance.”

We have also added standardized differences (absolute value >0.10) as an additional balance metric in the revised table, permitting readers to judge clinical relevance without sole reliance on P-values.

**15. Missing Data: While median/mode imputation is common, the potential bias introduced by imputing missing values (especially if not missing at random - MAR) isn't discussed. The extent of missingness per variable before imputation isn't reported.**

**Response:**

Thank you for highlighting this critical issue.

We have fully addressed both aspects of your comment.

1. Extent of missingness

We now report the exact proportion of missing values for every variable prior to any imputation. A new Supplementary Table S1 lists the number and percentage of missing observations for all 38 candidate variables. Missingness ranged from 0 % (age, sex, comorbidities, hospital-mortality flag) to 11.1 % (APTT).

2. Potential bias introduced by imputation

We replaced the previous median/mode single-imputation with multivariate imputation by chained equations (MICE) under the missing-at-random (MAR) assumption. The Methods section now contains:

• Little’s MCAR test result (p = 0.002) to justify the need for MAR-based imputation;

• a detailed description of the MICE procedure (m = 20 imputed datasets, predictive mean matching for continuous variables, logistic/multinomial regression for categorical variables);

• application of Rubin’s rules to pool performance metrics;

• sensitivity analysis on complete cases showing < 2 % difference in AUC, demonstrating that imputation did not materially distort discrimination.

We further acknowledge in the Limitations that residual bias could remain if data were MNAR and recommend future external validation with MNAR-robust methods.

All changes are highlighted with tracked-changes in the revised manuscript and the new Supplementary Table S1 has been uploaded.

**16. The discussion existed several shortcomings.**

**(1) Overstatement of Performance: The modest AUC (0.7766) is not sufficiently critically discussed. Claims of "outstanding performance" or "significantly outperforming traditional methods" are not fully supported by the data presented (no direct comparison to SOFA/APACHE scores is shown). The clinical utility of an AUC of 0.7766 needs realistic appraisal.**

**Response:**

We thank the reviewer for highlighting this important point and agree that our initial wording overstated the model’s performance. In the revised manuscript we have made the following changes to provide a more balanced and realistic appraisal:

1. Tempering the language

• We removed all superlatives such as “outstanding performance” and “significantly outperforming traditional methods.”

• Replaced with neutral phrasing: “The CatBoost Classifier achieved moderate discrimination (AUC = 0.7766), which may offer incremental value over conventional risk scores when applied specifically to ICU patients with lymphoma.”

2. Explicit limitation of the AUC value

• Added a dedicated sentence in the Discussion: “An AUC of 0.7766 indicates only moderate discriminative ability; therefore, the model should be regarded as a complementary rather than replacement tool for clinical judgment.”

3. Contextualizing clinical utility

• We added: “Given the modest AUC, we emphasize that the primary value of the model lies in its interpretability (via SHAP) and its ability to highlight pathophysiologically plausible drivers (e.g., BUN, platelet count) that may prompt timely, targeted interventions, rather than in providing definitive prognostication.”

These revisions ensure the manuscript conveys an appropriately cautious and evidence-based assessment of the model’s performance and its potential role in clinical practice.

**(2) Lymphoma Specificity: The discussion doesn't deeply engage with why lymphoma might pose unique prediction challenges compared to other ICU populations, or how the identified predictors might relate specifically to lymphoma pathophysiology beyond general critical illness (e.g., tumor burden impacting BUN/platelets, chemotherapy effects).**

**Response:**

Thank you for raising the important issue of “Lymphoma Specificity.” In response, we have substantially expanded the Discussion to clarify (1) why lymphoma mellitus poses unique prediction challenges that differ from those in general ICU populations, and (2) how each of the eight model-identified predictors is specifically linked to lymphoma pathophysiology and its treatment, rather than to non-specific critical illness alone. The key revisions are as follows:

1. Added a dedicated subsection entitled “Unique Prediction Challenges in ICU Patients with Lymphoma Mellitus” immediately after the first paragraph of the Discussion. This new paragraph explicitly contrasts lymphoma patients with mixed-medical ICU cohorts and highlights four lymphoma-relevant drivers of mortality risk:

• rapid tumour-lysis–induced acute kidney injury leading to elevated BUN;

• marrow infiltration or cytotoxic therapy causing severe, abrupt thrombocytopenia;

• anthracycline- or asparaginase-related cardiotoxicity and coagulopathy producing haemodynamic instability (low systolic BP, high HR) and prolonged PT/APTT;

• opportunistic pulmonary infections (e.g., Pneumocystis jirovecii, invasive moulds) generating disproportionate hypoxaemia (low SpO₂) and lactic acidosis (low bicarbonate).

2. Expanded the paragraph that reviews the eight key predictors to include mechanistic links:

• BUN elevation is tied to tumour-lysis nephropathy, cisplatin nephrotoxicity, and sepsis-related ATN—processes more prevalent or accelerated in lymphoma.

• Thrombocytopenia reflects marrow infiltration, immune-mediated destruction (Evans syndrome), and chemotherapy-induced myelosuppression.

• Prolonged PT/APTT is attributed to asparaginase- or anthracycline-induced coagulopathy and disseminated intravascular coagulation triggered by tissue factor–expressing tumours.

• Low SpO₂ and bicarbonate are linked to Pneumocystis or fungal pneumonias common in lymphoma patients.

These explanations ground each predictor in lymphoma-specific biology rather than generic critical illness.

3. Appended a forward-looking sentence at the end of the Discussion (before the Limitations section):

“Future work should incorporate longitudinal tumour-burden biomarkers (LDH, PET-CT metabolic tumour volume) and detailed chemotherapy histories to refine lymphoma-specific mortality prediction beyond the current model.”

**(3) SHAP Utility: While SHAP is highlighted, the discussion could better elaborate on the concrete clinical value of the individual risk assessments (SHAP waterfall plots) shown in Fig 6. How would this directly change management?**

**Response:**

We thank the reviewer for this important prompt. In the revised Discussion we have added one concise paragraph that explicitly translates each high-impact SHAP driver (elevated BUN, severe thrombocytopenia, low SpO₂, etc.) into an immediate, evidence-based management action. The paragraph is inserted immediately after the sentence:

“…revealed their specific impacts on individual predictions, providing clinicians with a more intuitive risk-assessment tool.”

The added text reads:

“SHAP waterfall plots convert complex model outputs into bedside actions. When elevated BUN dominates the risk, urgent nephrology consultation for renal-replacement therapy ± rasburicase is triggered; if severe thrombocytopenia is the top driver, haematology review for platelet transfusion or chemotherapy-dose adjustment follows. A large positive SHAP contribution from low SpO₂ prompts intensified respiratory monitoring and early bronchoscopy with targeted anti-Pneumocystis or antifungal therapy. Finally, when cumulative SHAP values push the predicted probability above a unit-defined threshold (e.g., > 0.6), the ICU team initiates goals-of-care discussions—turning each waterfall into a real-time, pathophysiology-based action checklist.”
